# Miscellany

**Published:** 1902-11

**Authors:** 


### MISCELLANY.

### Dr. Bissell's Laboratory Course.

DR. WILLIAM G. BISSELL, pathologist to the department of health, announces that the first session of this year's course in bacteriology will be given in the laboratory of the health department, Tuesday evening, November 3, 1902, at 8.15 p.m. The course will cover a period of six weeks' instruction, two evenings (Tuesday and Friday) each week.

Bacterial diagnosis of pus, sputum, secretions, membranes, gonorrheal discharge, typhoid blood, etc., will be covered, together with the manufacture of culture media, the preparation of cultures, staining of bacteria, and the like.

Those who desire to avail themselves of this opportunity, are requested to communicate with Dr. Bissell at once. Fee, $15 in advance, which covers all expenses.

DR. JOHN T. PITKIN, of Buffalo, announces that he has installed at his Ellicott Square office the actinolite or Finsen apparatus.

THE Arlington Chemical Company, of Yonkers, has published in pamphlet form a series of short lectures by Rev. Andrew F. Underhill, rector of St. John's Episcopal Church, of Yonkers, N. Y., entitled, Valid objections to so-called christian science. This handsome brochure is being addressed to all physicians. If any of our readers have been overlooked in the mailing, a request to the above-mentioned Company will bring a copy.

